# Root canal curvature as a prognostic factor influencing the diagnostic accuracy of radiographic working length determination and postoperative canal axis modification: an in vitro comparative study

**DOI:** 10.1186/s12903-021-01446-x

**Published:** 2021-03-02

**Authors:** Bestoon Mohammed Faraj

**Affiliations:** College of Dentistry, Conservative Department, University of Sulaimani, Madame Mitterand Street 30, 46001 Sulaimani, Kurdistan Region Iraq

**Keywords:** Canal curvature, Cone‐beam computed tomography, Canal axis, Digital periapical image, Real canal length

## Abstract

**Background:**

Radiographic analysis of tooth morphology is mandatory for accurate calibration of the degree of canal curvature angle and radiographic working length to its real dimensions in case difficulty assessment protocols. This study aimed to determine the impact of the degree of root canal curvature angle on maintaining the real working length and the original canal axis of prepared root canals using a reciprocating rotary instrumentation technique.

**Methods:**

Radiographic image analysis was performed on 60 extracted single-rooted human premolar teeth with a moderate canal curvature (10°–25°) and severe canal curvature (26°–70°). Working length and longitudinal canal axis were determined using cone-beam computed tomography (CBCT) and digital periapical radiography. The real canal length was determined by subtracting 0.5 mm from the actual canal length. Root canals were prepared using the WaveOne Gold reciprocating file (Dentsply Maillefer, Ballaigues, Switzerland).

**Results:**

There was no significant relation of the degree of canal curvature angle to the accuracy of radiographic working length estimated on CBCT and digital periapical radiographic techniques (P > 0.05). Postinstrumentation changes in the original canal axis between moderate and severe canal curvature angles, assessed on CBCT and periapical digital radiographic images were statistically non-significant (P > 0.05).

**Conclusions:**

A standardized digital periapical radiographic method performed similarly to the CBCT technique near to its true working length. No significant interaction exists between the diagnostic working length estimation, postoperative root canal axis modification, and the degree of canal curvature angle, using reciprocating rotary instrumentation technique.

## Background

Root canal cleaning and shaping is a fundamental step in clinical endodontics, and it is directly related to subsequent disinfection and filling procedures. It must enhance a funnel shape from the cervical to the apical portion, preserving the preoperative canal curvature angle and the location of the major apical foramen [1, 2]. These technical objectives are of great significance and have always been much more challenging in curved root canals. Uncentered and extensive canal axis modification can result in iatrogenic errors such as strip perforation, ledge formation, and insufficient disinfection of prepared root canals [3, 4]. However, the use of rotary nickel-titanium (NiTi) files has improved the overall shaping outcome and reduced the possibility of procedural errors with a subsequently lower incidence of canal curvature straightening [5].

Root canal working length determination is a fundamental component of the endodontic examination and treatment planning procedure. Conventional 2-dimensional (2D) radiographs provide a cost-effective, high-resolution image, which continues to be the most commonly used method in daily restorative dental practice. However, intraoral radiography has some limitations because of its 2-dimensional nature, information may be difficult to interpret, especially in challenging conditions when the anatomy of the root canal system is complex [6, 7].

The validity of the working length plays a fundamental role in determining the success of root canal treatment and could be a predictor of possible complications [8]. Measurement of the working length may be much more difficult in curved canals, which makes measuring difficult [9]. Overestimation of the working length may give rise to over instrumentation beyond the apical foramen, and as a consequence, pain and discomfort can occur, whereas underestimation of the working length may result in incomplete root canal cleaning and shaping [10, 11].

A better outcome of root canal treatment achieved when the original canal axis is preserved and without creating iatrogenic events, such as instrument fracture, canal transportation, ledges, or perforations [12]. The evaluation of changes in canal shape after instrumentation has been suggested as a reliable process to assess the ability of a shaping technique to preserve the original axis of the root canal in its place [13].

In clinical endodontics, several technical parameters were applied to investigate the canal shape before and after radicular dentine instrumentation. The quantitative evaluation of postoperative root canal changes can be estimated by the “centering ratio” method on original root canal curvature angle [14, 15], or by measuring pre-and postoperative radicular dentine thickness, on superimposing radiographic images before and after shaping using CBCT scanning or digital radiographic imaging techniques[16, 17]. Furthermore, analysis of change in canal curvature angle after shaping has been widely used in the literature to evaluate the tendency of a technique, or the mechanical properties of an instrument, to maintain the preoperative root canal geometry or to straighten the curves [18, 19].

WaveOne Gold (Dentsply Maillefer, Ballaigues, Switzerland) instrument is a single-file system with a reciprocating motion manufactured using a post-manufacturing thermal process that produces a file with superelastic nickel-titanium metal properties. This improvement in mechanical characteristics exhibits a unique alternating off-centered parallelogram-shaped cross-section and a progressively decreasing percentage taper design. The Primary WaveOne Gold instrument is more resistant to cyclic fatigue, more flexible with a better cutting efficiency than the conventional Primary WaveOne file [20, 21]. Several studies concluded that canal shaping using a single instrument with different kinematics did not compromise canal cleanliness and took less working time to prepare the canals compared with the conventional multi-instrument systems [22, 23].

Cone-beam computed tomography (CBCT) is a useful device that produces multiplanar reformatted images and allows two-dimensional views in all three dimensions (axial, coronal, and sagittal planes). It has been used as a research tool for various aspects of endodontics, such as analysis of angulation of root canal curvature and the efficacy of various instrumentation techniques on the outcome of shaping. Cone-beam computed tomography is confirmed to be more accurate than conventional x-rays in determining root canal systems, as well as have an impact on treatment planning [24, 25].

No clear evidence in the literature exists links quantity evaluation of canal curvature with working length estimation and shaping ability of reciprocating rotary instrumentation procedure. Although several studies conducted and assessed the association of instrumentation outcomes with many different factors, including the degree of canal curvature, and preoperative working length estimation. The experimental study of each particular parameter, in a curved canal, on extracted teeth, has yet to be precisely addressed, particularly in case difficulty assessment protocols. This study aimed first to investigate the influence of the degree of root canal curvature on the radiographic working length estimation on extracted single-rooted human premolar teeth. Secondly, to determine a link between the degree of root canal curvature angle and the postoperative change in longitudinal root canal axis, using a reciprocating rotary instrumentation technique through a quantitative evaluation, under the scrutiny of CBCT and digital radiographic imaging.

## Methods

### **Ethical approval and sample selection**

 The ethics protocol for this study was confirmed and accepted by the Ethical Committee at Sulaimani University (Protocol Number; 392/2020). The methodology applied has followed the CRIS guidelines as considered in the 2014 concept note [26]. A total of 60 curved single-rooted human premolar teeth with varying degrees of root curvature were selected for this study. The teeth with uncommon extreme variations were excluded, like twisted or fused roots. Endodontically treated teeth, internal or external root resorption were also excluded. Whereas those with completely formed apices, single canal with one apical foramen, and ≥ 10° canal curvature were included. The patients’ age, gender, tooth’s quadrant, or reason for extraction were not documented. Specimens were stored in 10 % formalin for disinfection for a maximum of 2 weeks. Tissue fragments and calcified debris were removed manually by using a hand scaler, then washed in running water. Finally, they were stored in normal saline at room temperature until the time of the investigation.

### Pre‐instrumentation digital periapical imaging

Each tooth was embedded in the radiolucent polysiloxane putty dental impression material (3 M ESPE, St. Paul, MN, USA), and encoded with a number. The digital radiographical examination was carried out for all the teeth in two directions (buccolingual and mesiodistal), using a standardized parallel technique. A high-frequency oral x-ray machine (EzRay Air W; Vatech, Korea), were used with an exposure time of 0.367 seconds (60 kV, 4 mA). The target– receptor distance was increased to compensate for image magnification and to ensure that only the most parallel rays were directed toward the tooth and the X-ray sensor (EzSensor Classic, Vatech, Korea). As a result, a long (16-inch) target–receptor distance was used [27].

### Pre-instrumentation CBCT scans

Two custom-made wood boxes were used for the mounting of the teeth and to confirm the standardization for the CBCT images. Each tooth was embedded in cold-cure clear acrylic resin (Vertex Castavaria, Netherland) with a technical specification of 9 minutes dough time and 6 minutes working time at 55°C, using a cylindrical plastic container .Thereafter, they quoted with MS3 master die separator (Ivoclar Vivadent, USA) to enable precise repositioning during pre and post-instrumentation scans. Ten teeth were mounted in each template consistently by using dental stone plaster (Rident, Rajasthan, India). Each mold was horizontally fitted to the chin support of the CBCT machine (NewTom, Giano, Verona, Italy) in a way that the occlusal plane was arranged parallel to the plate, and scanned with 90 kVp, 3 mA, voxel size: 0.125 mm, exposure time: 5.4 s, by using FOV 8 cm by 11 cm [27, 28].

### Pre-instrumentation working length estimation on CBCT and digital periapical radiographic images

 Traceable calibration was performed in the center of the pulpal cavity and followed each visible canal deviation, thus allowing for the measurements of curved canals. The radiographic tooth length was determined on the CBCT and digital periapical images as the distance between the tip of the cusp and the major apical foramen. The radiographic working length for all the specimens was measured separately on digital periapical and CBCT images after subtraction of 1mm from the radiographic tooth length [27].

### Real working length measurement on extracted teeth

A standard straight-line access opening was prepared for all the teeth. A size #10 or #15 K-File (Dentsply Maillefer, Ballaigues, Switzerland) was passively advanced until its tip was seen at the level of the coronal most boundary of the major apical foramen, by the aid of a magnifying lens (Keeler, Windsor, UK, ×3 magnification). The distance between the reproduced coronal reference point and the tip of the file was measured with an electronic digital caliper (Mitutoyo Corp., Japan) to the nearest (0.01 mm.), and documented as the actual working length. The real working length was determined by subtracting 0.5 mm from the actual canal length [27].

### Canal curvature measurements

An experienced oral and maxillofacial radiologist obtained all the CBCT and digital radiographic images and performed the measurements. The change in canal axis was determined as the difference between canal curvature before and after instrumentation. For the CBCT evaluation, scan images from the clear sagittal view were selected depending on the multiplanar imaging-reformatted sections. The slices were first reproduced in a vertical position to visualize the tooth cusp, pulp chamber, apical foramen, and the complete view of the root canal pathway. All images converted for viewing with image analysis software (NNT Software, Verona, Italy) to measure the canal curvature angle.

**Fig. 1:**
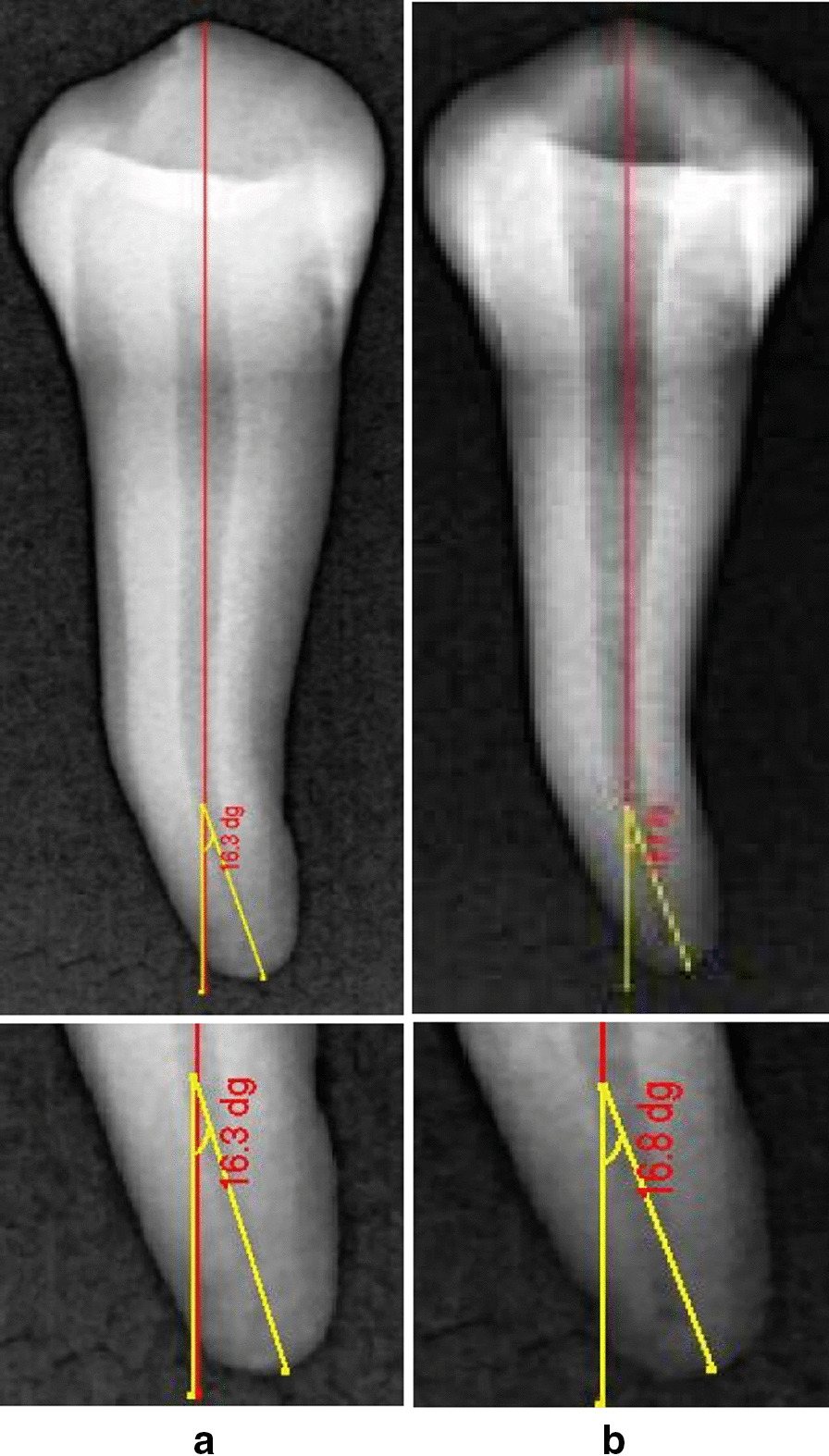
Representative digital periapical images **a** pre-instrumentation canal curvature angle measurement **b** post- instrumentation canal axis modification

**Fig. 2:**
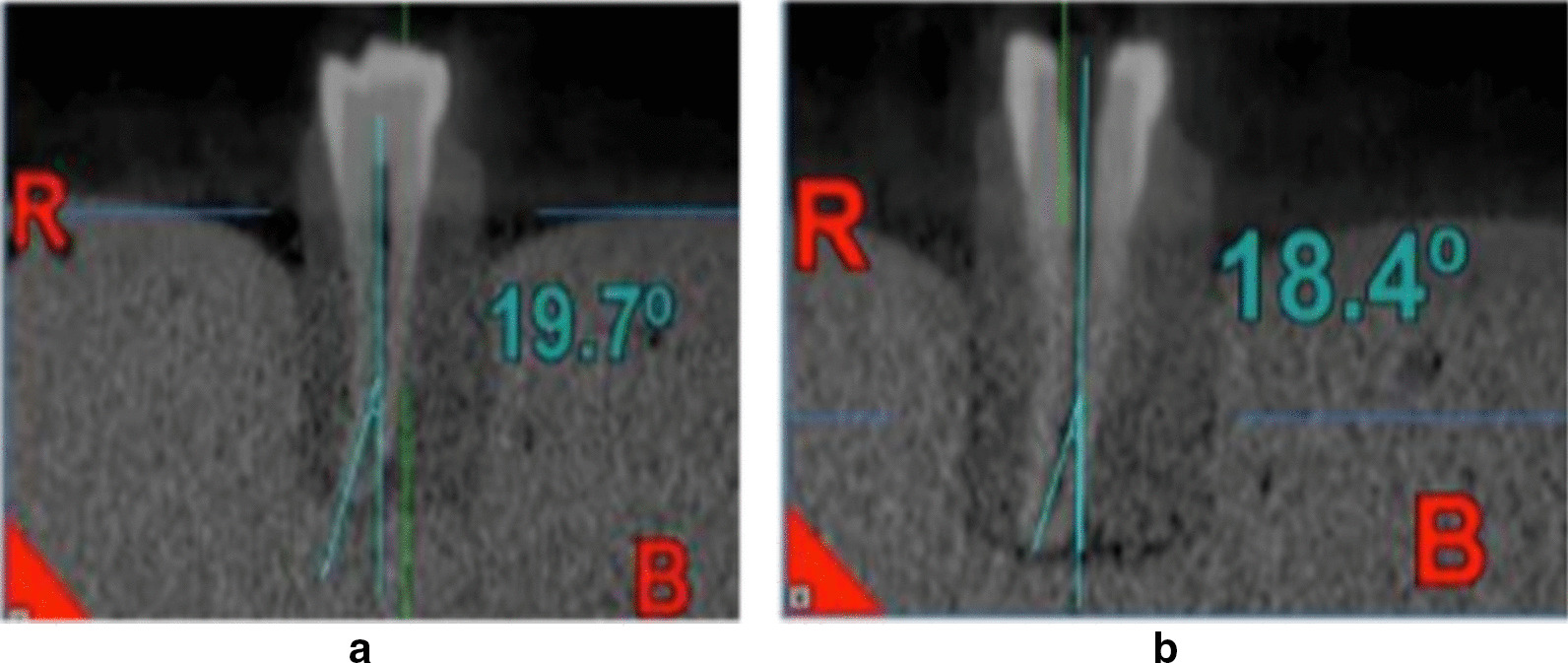
Representative CBCT images demonstrating a post-instrumentation canal curvature angle measurement

The Schneider method was applied for the estimation of the degree of canal curvature before and after instrumentation. Two straight lines of equal lengths were used. The first line represented the continuity of the apical region, and the second line followed the middle and coronal thirds of the root canal. The angle between the radii was geometrically measured, and the canal curvature was expressed in degrees (Figs. 1, 2).The formed canal angle was named according to the degree of root canal curvature into moderate (10-25°) and severe (26-70°). All scan images captured before and after instrumentation were analyzed with image analysis software (NNT Software, Verona, Italy), to determine the canal curvature changes [27, 29].

### Root canal cleaning and shaping

An endodontist, experienced in the use of the Primary WaveOne Gold Reciprocating file (DentsplyMaillefer, Ballaigues, Switzerland), performed all procedures. The analysis of the radiographic images reproduced in three dimensional for the canal curvatures and shaping ability was carried out by an oral and maxillofacial radiologist who was blind in respect of all experimental groups.

The access coronal cavity was prepared using a round carbide bur #4 (Dentsply Maillefer, Ballaigues, Switzerland), and the canal patency checked with a #15 K-type hand file (Dentsply Maillefer, Ballaigues, Switzerland). The coronal third flared with Gates-Glidden drills 2 and 3 (Dentsply Maillefer, Ballaigues, Switzerland). Working length was confirmed manually with a #15 K-File using a standard protocol. A glide path created using a ProGlider instrument (16/02) (Dentsply Maillefer, Ballaigues, Switzerland) carried to the working length. A handpiece generated by an electric motor (Silver, VDW, Munich, Germany) was used for instrumentation. The speed, torque, and file sequence were applied according to the manufacturer’s instructions. Only five canals were instrumented at each time interval to minimize operator fatigue. After each file sequence, the prepared canal was washed out with 3 ml of 3 % NaOCl solution (Techno Dent, Greece), followed by a 5 ml solution of %17 EDTA (SPIDENT, Korea). Then, the canals were irrigated with 5 ml of 3 % NaOCl as a final rinse. Root canal irrigations were performed by using a 5 mL disposable plastic syringe with a 30 gauge side opening needle (Optimus, SP, Brazil) at room temperature. The needle was inserted inside the canal without binding and the solutions were introduced slowly and passively allowing adequate back-flow.

### Post‐instrumentation CBCT and digital periapical image analysis

After instrumentation, each tooth was repositioned in its previous position inside a plaster block. The post-CBCT and digital periapical radiographs were obtained with the same parameter applied in the pre- instrumentation phase. Longitudinal axial canal axis and canal curvature angle were determined (Figs. 1, 2). The percentage of the change in canal curvature angle after instrumentation (canal axis modification) was calculated using the following formula [29];

### Canal curvature angle after instrumentation-canal curvature angle before instrumentation

Canal curvature angle before instrumentation X 100.

### Statistical analysis

Statistical analysis of data obtained in this study performed using IBM SPSS Statistics for Windows, version 24.0 (Armonk, NY: IBM) software. A P-value < 0.05 was considered a statistically significant level. The sample size was determined with the Sealed Envelope software for a power of 80 %. The normal distribution of the data was tested using the Shapiro-Wilk test. Chi-square tests were used to compare the frequencies of qualitative variables. When the distribution of variables was normal, an independent sample t-test was used to compare the results within the investigated parameters between moderate and severe curvature angles.

## Results

**Fig. 3:**
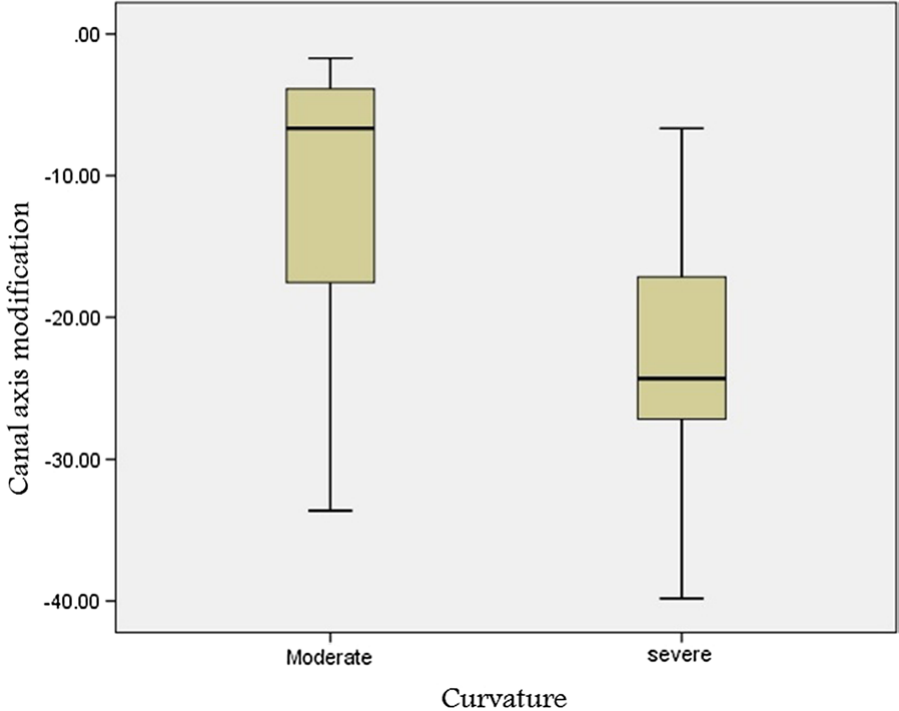
Box plots comparing the relation of the degree of root canal curvatures with the postoperative canal axis modifications assessed on CBCT images

The values of the mean and standard deviation of working canal length concerning the degree of canal curvature angle close to its real clinical canal length are summarized in (Tables 1, 2). There was no remarkable role of the degree of canal curvature angle on the precise estimation of radiographic canal length (P > 0.05). Regarding the CBCT image scanning, there was a tendency for underestimation of the working canal length from − 1.2mm to − 0.1mm (− 0.538 ± 0.303) in 52 (86.7%) of the examined specimens. Whereas, overestimation from 0.1mm to 2.0mm (0.7 ± 0.775) were reported in only 5 teeth (8.3 %). The periapical radiographic images slightly overestimated the real canal length measurement (0.635 ± 0.374). On the whole, this technique revealed an accuracy of 53.3 % (32), within the range of + 0.5 mm tolerance level (Table 2). The results from CBCT image scanning indicated an accuracy of 55% (33) within a range of + 0.5 mm tolerance level (Fig. 3).

**Table 1:** Comparison of mean and standard deviation values of working length estimated on CBCT and digital radiographic images in relation to the degree of canal curvature angles

		Degree of curvature Mean ± SD			P value *
		Moderate 10°–25°	Severe 26°–70°	Total %	
Digital image	Overestimation Closest to real length Minimun to Maximun N	20.86 ± 1.63 0.624 ± 0.409 0.2 to 2.3 43	20.43 ± 0.83 0.658 ± 0.293 0.3 to 1.2 17	20.72 ± 1.43 0.635 ± 0.374 0.2 to 2.3 60(100%)	0.27 ** 0.75**
CBCT image	Overestimation Closest to real length Minimun to Maximun N	19.43 ± 1.22 0.675 ± 0.892 0.1 to 2.0 4	20.3 ± 0.80 ± − 0.8 1	19.6 ± 1.13 0.7 ± 0.775 0.1 to 2.0 5(8.3%)	0.47** 0.91**
	Underestimation Closest to real length Minimun to Maximun N	19.83 ± 1.72 − 0.494 ± 0.306 − 1.0 to − 0.1 36	19.41 ± 1.02 − 0.638 ± 0.280 − 1.2 to − 0.2 16	19.70 ± 1.54 − 0.538 ± 0.303 − 1.2 to − 0.1 52(86.7%)	0.81** 0.12**

*Independent t test

**Not significant

**Table 2:** Estimation accuracy of working length measurement closest to real values in a range of 0.5mm tolerance level recorded on CBCT and digital radiographic images

Tolerance level		Degree of curvature		Total %	P value*
		Moderate 10°–25°	Severe 26°–70°		
CBCT image 0.5 mm	Yes	26 60.5%	7 41.2%	33 55%	0.18**
	No	17 39.5%	10 58.8%	27 45%	
Digital image 0.5 mm	Yes	25 48.8%	7 41.18%	32 53.3%	0.92**
	No	18 51.2%	10 58.82%	28 46.7%	

*Chi- square test

**Not significant

**Fig. 4:**
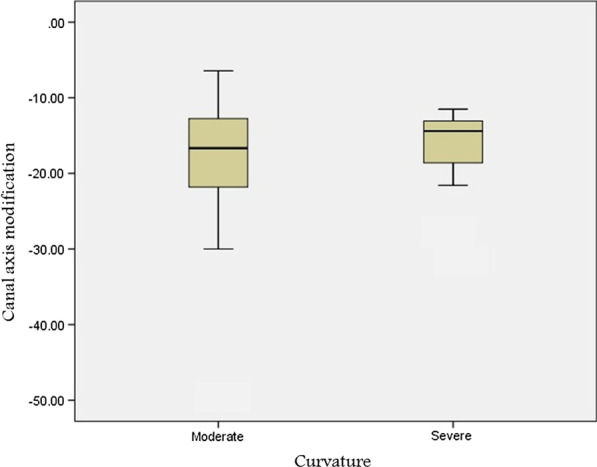
Box plots comparing the relation of the degree of root canal curvatures with the postoperative canal axis modifications assessed on digital periapical radiographic images

The canal preparation led to a decrease of the root canal curvature for both moderate and severe canal curvature angles collectively (Tables 3, 4). The difference between before and after instrumentation shows statistically no significant deviation of curvature between moderate and severe canal curvature angles reproduced on CBCT and digital periapical radiographic images (p < 0.75) (Figs. 3, 4).

**Table 3:** The mean and standard deviation (SD) values of the change in the canal curvature angles after shaping in both moderate and sever curvature groups, calculated on CBCT images

	Moderate 10°–25°			Severe 26°–70°			P-value*
	N	Mean	SD	N	Mean	SD	
Before instrumentation	43	21.32	4.26	17	29.95	4.143	0.688**
After instrumentation	43	18.541	4.143	17	22.84	2.693	0.1**
Curvature change	43	-12.721	10.016	17	-23.202	10.131	0.691**

* Independent t test

** Not significant

**Table 4:** The mean and standard deviation (SD) values of the change in the canal curvature angles after shaping in both moderate and sever curvature groups, calculated on digital periapical radiographic images

	Moderate 10°–25°			Severe 26°–70°			P-value*
	No.	Mean	SD	No.	Mean	SD	
Before instrumentation	43	18.24	4.06	17	28.85	2.13	0.013**
After instrumentation	43	15.62	3.82	17	24.75	2.14	0.023**
Curvature change	43	− 16.82	7.71	17	− 18.82	5.76	0.388***

*Independent t test

**Significant

***Not significant

## Discussion

In the present study, the correlation between the diagnostic working length calculations, the parameter of the shaping outcome (canal axis modification) in root canal preparation, and its degree of canal curvature angle were investigated under *ex vivo* conditions. Several techniques are available for the comparative evaluation of the preoperative radiographic working length determination and shaping outcome of reciprocating rotary Nickel-Titanium (NiTi) instruments in curved canals. The comparison of the pre-and post-instrumentation radiographic images of the outlines of the longitudinal root canal axis enabled the evaluation of the most relevant parameters of root canal preparation. Furthermore, assessment of their diagnostic validity and the possible influence of other associated factors such as degree of canal curvature can be performed using appropriate computer software [30].

The major advantage of the use of extracted teeth in experimental investigations is the reproduction of the clinical situation, an inspection of root canal morphology, and the establishment of a satisfactory real positive finding. A human premolar tooth with a single root canal was selected in the present investigation to eliminate variables specific to root canal configuration. However, it is difficult to standardize variables such as root canal configuration, and the nature of canal curvatures. The reliability, accuracy, and certainty of these study protocols had previously been approved in the literature reviews [31–33].

In the present methodology, the measurement of root canal curvature was calibrated by using CBCT scanning. This allows improved precision and helps to increase the evaluation accuracy of the cross-sectional shape of prepared root canals through a three-dimensional radiographic image with no destruction of the specimens [34, 35]. With this technique, it is feasible to compare the longitudinal canal axis of the root canal before and after shaping. Due to these considerations, the worksheet was made for only 60 teeth. Attempts were made to ensure the standardization of the selected teeth through several inclusion and exclusion criteria.

The findings of the present investigation validated the accuracy of the working length measurements conducted on radiographic images in various degrees of curvature, using a standardized paralleling technique, closest to the CBCT and real canal length values. In line with Connert et al. [36], calibration of canal length on CBCT and digital radiographic images were slightly more accurate when performed for root canals having a moderate canal curvature. While, there was no statistically significant difference in mean values of root canal length measured with an extensive degree of curvature angle, in comparison to its real length. These outcomes are most probably related to the direction and nature of root angulation and anatomic noise, which results in the missing of some details of the root canal anatomy assessed with the periapical digital radiography. However, CBCT scanning overcomes this distortion in image quality by maintaining the multiplanar reconstruction, which leads to a clear 3D visualization of the root canal configuration.

Primary WaveOne Gold reciprocating file was selected for this study since clear evidence in the literature exists to support its efficiency in the preparation of different root canal morphology [37–39].In the present study, we chose not to include conventional stainless steel K-files as a control group. The superiority of NiTi instruments in maintaining the original canal shape better than K-files is already established in the literature [40, 41]. The results from the present study confirmed the ability of rotary NiTi instruments to maintain the original canal curvature, even in extremely curved canals, and showed that none of the tested moderate and severe canal curvature samples reached the aforementioned critical level of canal straightening, keep its original canal axis. Similar findings were observed in previous studies performed on root canals with severe curvature [38, 39].The increased flexibility of NiTi instruments is behind their good centering ability and maintaining the original canal axis [40]. According to the manufacturer instructions, the technology applied in WaveOne Gold files expands the flexibility and strength of the instrument [42], as well as enhancing its elasticity and its reciprocating motion, which maintains the central canal path while shaping it and reduced the incidence of procedural errors [43, 44].

The results of the present study revealed that the use of the WaveOne Gold reciprocating file results in a higher mean value of the postoperative canal axis modification in teeth with severe canal curvature than moderate curvature. This outcome might be related to the fact that canal curvature may have caused the non-uniform distribution of stress on the instrument and increased transportation values [21–23]. Another promising finding was that the limited canal axis modification in this study might be related to the good centralization capacity of the instruments in the canal, especially in the apical- and middle third. The angle of the canal curvature was confirmed to be the most relevant factor affecting the outcome of canal shaping. The straightening of the canal during instrumentation occurs particularly in curved root canals [12].

The variation related to the study results in the literature may be due to the determination method of root canal curvature. In this study, after conventional access preparation and coronal flaring, straight-line access was established. Other factors that may have contributed to the minimal canal straightening observed in the study were operator-related. All teeth were prepared by an experienced operator in rotary instrumentation. The shaping procedure led to a decrease in the root canal curvature in all tested specimens. More specifically, more deviation in teeth with a severe degree of canal curvature than in the moderate canal curvature group. These results suggest that despite the advantages provided by the superelasticity of the rotary instruments, early coronal enlargement and the concept of straight-line access might enhance the shaping outcome in severely curved root canals with a minimum amount of iatrogenic errors. Future studies could fruitfully explore this issue further by evaluating different shaping parameters.

The sample size in this study was small, and the process of scanning and reconstructing radiographic images were limited to 60 teeth. Larger sample size will compensate for anatomical variables and may show if there are differences in findings regarding more complex canal morphology focusing on the number and classes of canal configuration. It is also important to be aware of possible sources of errors to avoid over interpretation of radiographic images. However, based on the protocol applied in this work for working length confirmation, the instrument prepared root canals to acceptable good clinical standards. The clinical relevance of radiographic working length confirmation to the degree of root canal curvature concerning the ultimate shaping outcome of instrumentation is likely to be critical in case difficulty assessment protocols.

## Conclusions

Under the limitations of this in vitro study and based on the applied radiographic working length image analysis, the present findings showed that a standardized digital periapical radiographic method performed similarly to the CBCT technique near to its real canal length. Calculation of the working length and maintaining post instrumentation canal curvature axis were not influenced significantly by the degree of canal curvature, using reciprocating rotary instrumentation technique.

## Data Availability

The datasets used and/or analysed during the current study are available from the corresponding author on reasonable request.

## Abbreviations

CBCT: Cone-beam computed tomography
FOV: Field of view
SD: Standard deviation
KVp: Kilovoltage peak
mA: Milliampere
2D: Two-dimensional
3D: Three-dimensional
